# Experimental Study on Three-Dimensional Microstructure Copper Electroforming Based on 3D Printing Technology

**DOI:** 10.3390/mi10120887

**Published:** 2019-12-17

**Authors:** Yuanyuan Wu, Shuangqing Qian, Hua Zhang, Yong Zhang, Hongbei Cao, Mingyu Huang

**Affiliations:** 1 School of Mechanical Engineering, Nantong University, Nantong 226019, China; wuu0901@outlook.com (Y.W.);; 2 Shazhou Professional Institute of Technology, Zhangjiagang 215600, China

**Keywords:** three-dimensional microstructure, electroforming, 3D printing, process parameters

## Abstract

In order to fabricate three-dimensional metal microstructures, a combined machining process based on 3D printing technology and electroforming technology is proposed. Firstly, a substrate with microstructures is fabricated by 3D printing technology, and then the microstructures were fabricated by electroforming technology. The influence of process parameters such as current density, distance between electrodes and pulse current duty cycle on the electroformed layer were studied and analyzed. It was determined that the peak current density 6A/dm^2^, the void ratio 20%, and the distance between electrodes 40 mm were the optimum process conditions of electroforming experiment. The electroforming experiments of different microstructures were carried out with the optimum process parameters.

## 1. Introduction

The microstructures on the surface of the components [1,2] make it able to obtain mechanical, electrical, optical, and other properties, so it is widely used in micro-mechanical electronics, new energy, aviation equipment, biomedicine, and other fields. Due to the good thermal conductivity, electrical conductivity and mechanical properties of metal materials, the machining technology of microstructures on a metal surface [3] has received extensive attention worldwide.

In recent years, the machining technology of microstructures mainly include electrochemical machining [4,5], electrical discharge machining [6], laser micromachining [7], the micro embossed molding method [8], and electroforming technology [9]. Rathod et al. [10] studied the influence of electrochemical micromachining parameters on machining accuracy during fabrication of a 500 μm-deep microgroove in stainless steel, and a high quality microgroove was fabricated using the optimum setting of machining parameters. Jafari et al. [11] fabricated metal-based micro-channel with different surface texture by micro-wire electrical discharge machining and found the optimum set of process parameters. Borghi et al. [12] used a laser micro-molding technique on the surface of 30NiCrMo12 nitriding steel to prepare array micro-pits with a diameter of 100 μm, a depth of 50 μm and an area ratio of 40%. Ehrhardt et al. [13] transferred micro-and submicrometer structures from nickel foils into solid copper surfaces by laser microembossing, and in the micrometer range the replicated patterns feature a high accuracy regarding the shape. Zhou et al. [14] presented a method to replicate metallic stamp via combined nanoimprint and electroforming. Structures with dimensions ranged from several microns to sub-100 nm were replicated in large patterned area. To fabricate sophisticated microstructures, Weinmann et al. [15] proposed a process chain combining photolithography, electroforming and pulse electrochemical machining (PECM). They fabricated the microstructured part by photolithography and electroforming, which can be used as a tool in the PECM process. Ming et al. [16] propose a hybrid electrochemical fabrication process combining electroforming and mask electrochemical machining to manufacture metal through-hole arrays with double tapered openings. Favorable through-holes featuring good opening profile symmetry and smooth surfaces was machined using this hybrid process.

With the development of micro-structure application, its machining technology is also constantly developing. Electroforming is a special machining technology that accurately replicates the same products as prototypes by electrodeposition. Electroforming can convert the inner surface that is difficult to be machined to the outer surface of the core mold and accurately replicates the surface contour and fine structure. It has excellent replication and repeatability. Therefore, Electroforming is widely used in aerospace, precision molds, weapon manufacturing, and other fields. 3D printing is a rapidly developing technology in the manufacturing industry. Its biggest advantage is that it can complete the production of various shapes without mold. In this paper, the combination of 3D printing technology and electroforming technology was used to fabricate three-dimensional metal microstructures. This processing method can simplify the process of cathode preparation and shorten the processing time. The influence of different machining parameters like current density and distance between electrodes on the microhardness and surface morphology of the electroformed layer were studied. Based on this, the pulsed current was used for the electroforming experiment. The influence of different duty cycles on the electroformed layer was analyzed and the optimal machining parameters for electroforming experiment were obtained. The three-dimensional microstructures with good forming quality were finally fabricated.

## 2. Experimental Details

**Figure 1:**
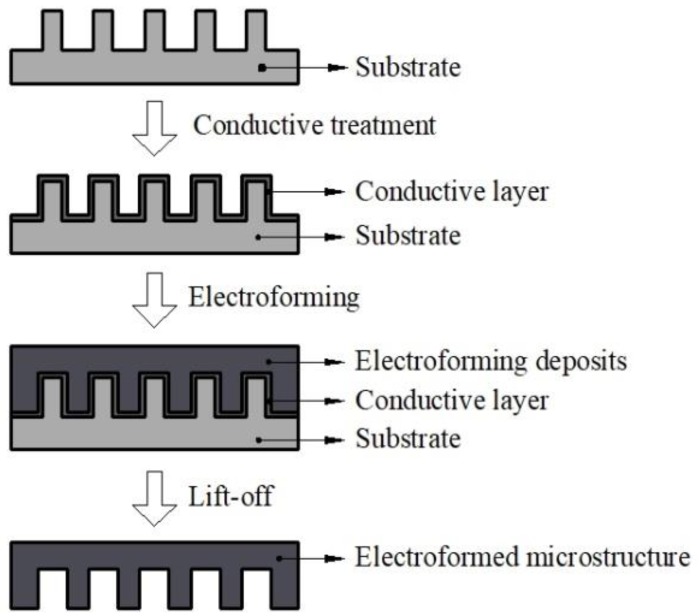
Outline of the steps involved in fabricating the microstructure using 3D printing and electroforming technology.

The outline of the steps involved in fabricating the microstructure using 3D printing and electroforming technology is shown in Figure 1. Firstly, a three-dimensional model with microstructures was built by three-dimensional modeling software, and a non-metal substrate was fabricated by 3D printing technology. Then a conductive layer of silver paint was coated on the surface of the non-meta substrate. After the electroforming process was completed, the sample was put into the laboratory oven for 10 min to soften the substrate so that it could be easily separated with the electroformed layer. A metal plate with three-dimensional microstructures was obtained.

**Figure 2:**
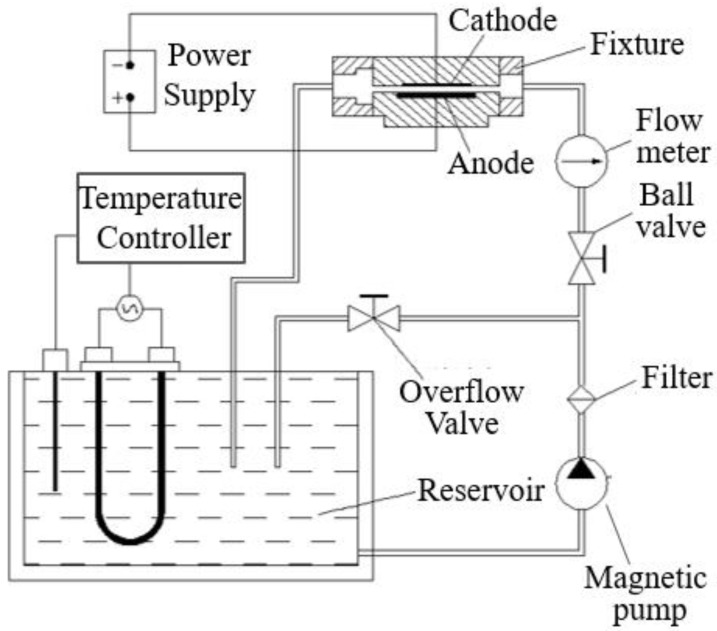
Schematic diagram of the electroforming equipment.

**Figure 3:**
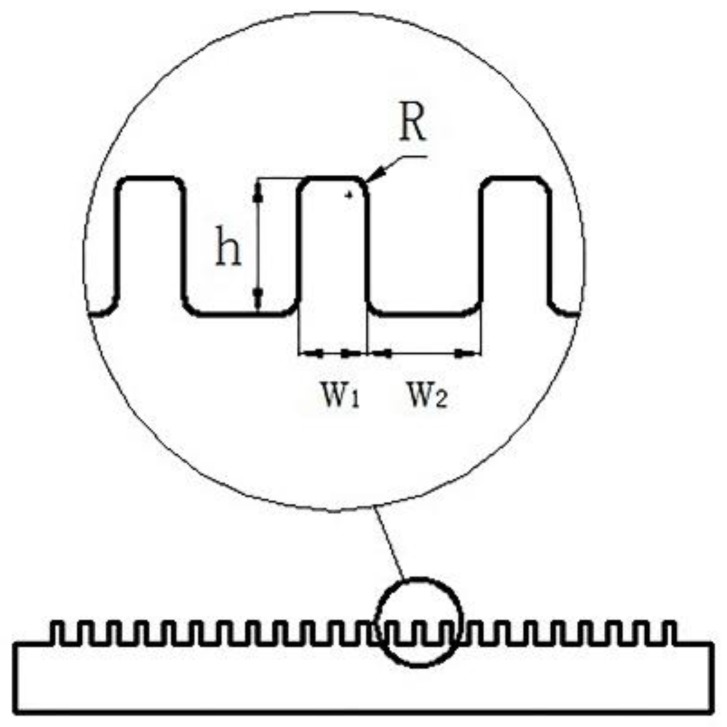
Micro-grooves.

**Figure 4:**
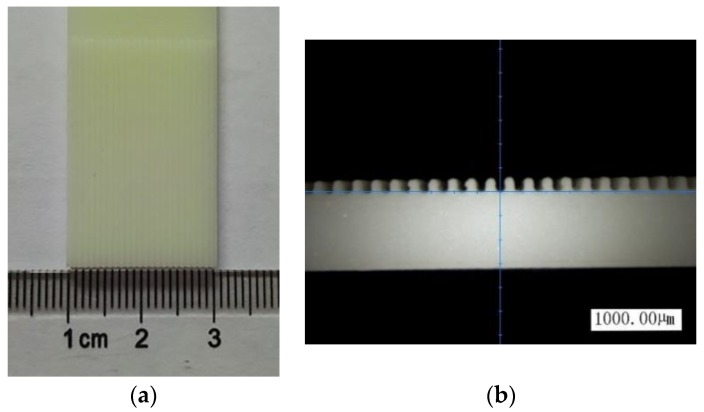
Substrate fabricated by 3D printing technology. (**a**) substrate and (**b**) cross-section of the substrate.

The electroforming equipment mainly includes a power source, a temperature controller, a reservoir, an electrode fixture, etc., as shown in Figure 2. As the central part of the electroforming equipment, the power source was used to adjust the current density of the electroforming and it can provide direct current and pulse current. The temperature controller was used to keep the electrolyte at a certain temperature. The flow meter, ball valve, filter magnetic pump, and overflow valve were used to circulate the electrolyte solution. The cathode and anode were fixed on the fixture. The anode was a phosphor bronze plate and the cathode was made of ABS-like plastic. The ABS-like plastic could be softened at the temperature of 60 °C, which could make it easy to remove from the electroformed layer. Take micro-grooves as an example, its structure is shown in Figure 3, wherein h = 600 μm, w_1_ = 300 μm, w_2_ = 500 μm, and R = 50 μm. The substrate was fabricated by a LITE 1000 3D printer, which was based on stereo lithography appearance (SLA). The processing method of SLA was focusing ultraviolet light with a specific wavelength and intensity on the surface of the material, and solidifying it from point to line and from line to surface in order to complete the drawing of a layer section. In this way, the printing of a three-dimensional solid layer by layer was completed. The substrate is shown in Figure 4a and the substrate with conductive layer is shown in Figure 4b.

The electrolyte solution of copper electroformed is the acidic copper sulfate solution, which is simple and stable. The composition of the electrolyte solution and condition are shown in Table 1. After the electroforming experiment was completed, the electroformed layer was separated with the substrate and cut into 5 mm × 5 mm pieces by wire cut electrical discharge machining (WEDM). The pieces were mounted into metallographic mosaic, then rough ground, fine ground and polished. The microhardness of the electroformed copper layer was measured by the TMVS-1 digital microhardness tester from Beijing Time High Technology Co., Ltd. (Beijing, China). The measurement method of microhardness was to press a diamond indenter with a certain geometric shape on the surface of the piece with a small load of 0.981 N, and then optically measured the two diagonal lines of the indentation. The surface morphology of the electroformed copper layer was observed by the JSM-6510 scanning electron microscope from JEOL (Tokyo, Japan).

## 3. Results and Discussion

### 3.1. Influence of Current Density on the Electroformed Layer

**Figure 5:**
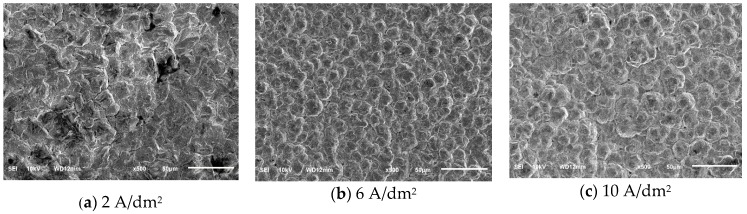
SEM images of electroformed surface under several current densities.

During the electroforming process, the surface morphology of the electroformed layer mainly depended on the nucleation rate and growth rate of the grain. When the nucleation rate was higher than the growth rate, the grain of the electroformed layer was refined. When the growth rate was higher than the nucleation rate, the grain size became coarser and the surface of the electroformed layer became rougher. The current density of cathode affected the nucleation rate and growth rate of the grains directly. Figure 5 is the SEM images of the surface of the electroformed copper layer, which was obtained under several current densities. Under the condition of distance between electrodes of 40 mm, with the increase of current density, the Cu grains on the surface of the electroformed copper layer was refined and then coarsened. Under a current density of 6 A/dm^2^, the surface of the electroformed copper layer was most uniform and dense. When the current density was low, the deposition rate was slower. At this time, the growth rate of the Cu grains was faster than the nucleation rate. When the current density was increased to 6 A/dm^2^, the nucleation rate of the Cu grains increased as the cathode overpotential increased, and the critical radius of the grains was smaller, thereby refining the Cu grains of the electroformed copper layer. However, when the current density was too large, the grains would become coarser, which could be seen in Figure 5c.

**Figure 6:**
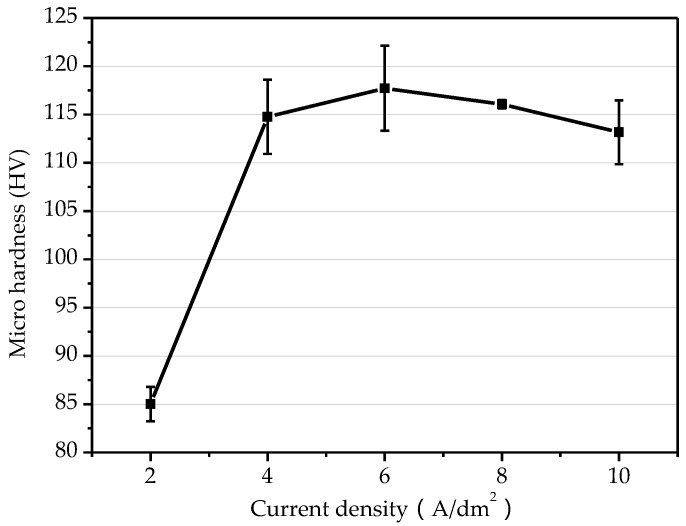
Influence of current density on microhardness.

Figure 6 shows the variation of the microhardness on the surface of the electroformed copper layer under current density of 2–10 A/dm^2^. When the current density was 2–6 A/dm^2^, the microhardness of electroformed copper layer increased with the increase of current density. The highest micro hardness value was 117.72. As the current density was gradually increased from 6 to 10 A/dm^2^, the microhardness of the electroformed layer tended to decrease. The mechanical properties of metal materials were related to the grain size and density. The strength value and grain size d usually obeyed the Hall–Petch relationship [17]:

(1) $$\sigma_{s}=\sigma +k\cdot d^{-1/2},$$

where: σ_s_ is the yield or elastic limit stress, σ is the lattice frictional resistance generated when moving a single dislocation, k is the Hall-Petch coefficient, and d is the grain size.

According to formula (1), there is a geometric inverse relationship between the strength of the material and the grain size, which means the smaller the grain size of the electroformed layer, the higher its strength. The increase of the current density within a certain range will accelerate the rate of grain formation and refines the grain, therefore improved microhardness. When the current density is too large, the rate of electrochemical reaction becomes faster, and the replenishment rate of metal ion is relatively slow. At this time, the nucleation rate of the grains is slower than its growth rate, resulting in formation of coarse grains and reduced microhardness.

### 3.2. Influence of Distance Between Electrodes on the Electroformed Layer

**Figure 7:**
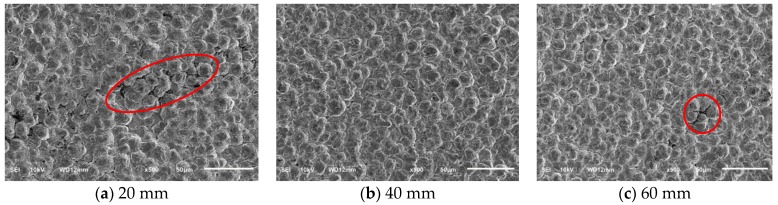
SEM images of electroformed surface under several distances between electrodes.

The SEM images of the surface of the electroformed layer at different distances between electrodes are shown in Figure 7. At a current density of 6 A/dm^2^, the grains of the electroformed layer were relatively uniform at a distance between electrodes of 40 mm. When the distance between electrodes was 20 mm and 60 mm, cracks occurred in the surface of the electroformed layer, which are shown in Figure 7, and the surface quality was poor. The distance between electrodes determines the resistance of the electrolyte solution between the anode and the anode, and the resistance affected the current on the surface of the cathode, which might cause the generation of internal stress. The internal stress was the main cause of crack formation.

**Figure 8:**
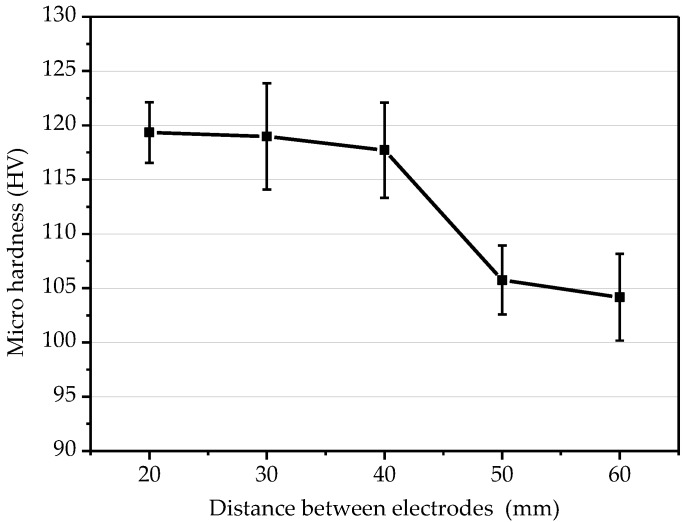
Influence of distance between electrodes on microhardness.

Figure 8 illustrates the influence of the distance between electrodes on the microhardness of the electroformed layer. Under the condition of the current density of 6 A/dm^2^, as the distance between electrodes increased, the microhardness of the electroforming layer decreased. When the distance was 60 mm, the microhardness was reduced to a minimum. At the same current density, the distance between the anode and the cathode had a certain influence on the current density distribution on the cathode surface. When the distance between electrodes was small, the current on the cathode surface was relatively high, and the nucleation rate of the grains was faster, so the electroformed layer had a high microhardness.

### 3.3. Influence of Duty Ratio on the Electroformed Layer

**Figure 9:**
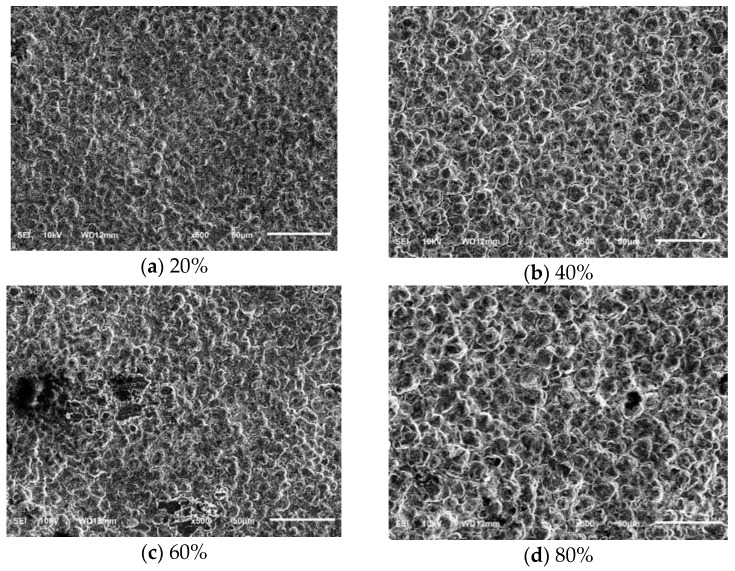
SEM images of electroformed surface under several duty ratios.

The SEM images of the surface of the electroformed layer at a different duty ratio are shown in Figure 9. When the pulse frequency was 2 H_Z_, the peak current density was 6 A/dm^2^, the distance between electrodes was 40 mm, the surface quality of the electroformed layer was significantly improved compared with the DC current. When the duty ratio was 20%, the surface of the electroformed layer was most uniform and dense. When the duty ratio was 60%, nodules and burrs appeared on the surface of the electroformed layer. When the duty ratio was 80%, the grains of the electroformed layer were obviously enlarged. This was because the larger the duty ratio means the longer the pulse time and the shorter the gap time, the higher the polarization of the metal ion concentration in the electrolyte and the concentration of the cathode surface, then it thickens the ion diffusion layer on the cathode surface. The smaller the duty ratio, the smaller the concentration polarization, the thinner the ion diffusion layer, and the electroforming process could be carried out at a higher overpotential. At this time, the nucleation rate of grains increased, resulting in the formation of fine grains on the surface of the electroformed layer.

**Figure 10:**
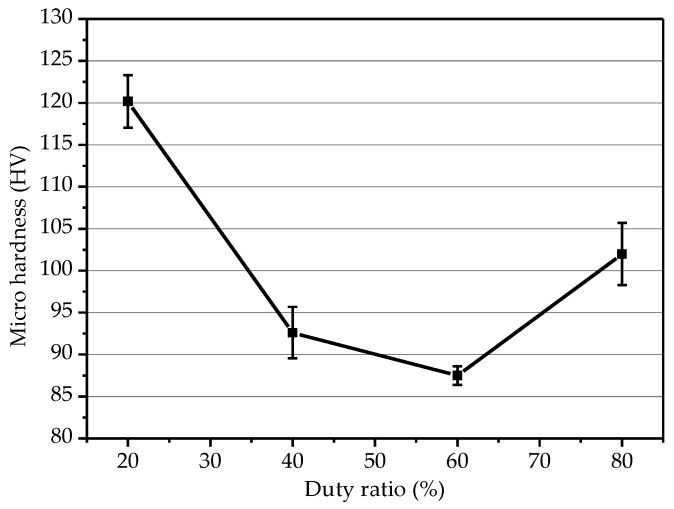
Influence of the duty ratio on microhardness.

Figure 10 is a graph showing the influence of different duty ratio on microhardness. Under the conditions of peak current density of 6 A/dm^2^, distance between electrodes of 40 mm, the microhardness of the electroformed layer decreased first and then increased with the increase of duty cycle, and the microhardness reached a maximum at a duty ratio of 20%. At high current density, as the duty ratio decreased, the thickness of the fine grain region of the electroformed layer increased, the grain size became smaller, and the microhardness was higher. In this paper, the microhardness obtained under the duty ratio of 80% was greater than the duty ratio of 40% and 60%, which was mainly due to many factors affecting the electroforming process. The influence of each factor was not independent and the interaction was complicated. Generally, electrochemical polarization and concentration polarization exist simultaneously during electroforming, and the cathode overpotential can be expressed as [18]:

(2) $$\eta_{c}=\frac{\mathrm{RT}}{\mathrm{nF}}\ln\frac{J_{K}}{J_{0}}+\frac{\mathrm{RT}}{\mathrm{nF}}\ln\left(\frac{J_{d}}{J_{d}-J_{K}}\right),$$

where: J_0_ is the exchange current density, J_d_ is the ultimate diffusion current density, J_K_ is the cathode current density, R is the molar gas constant, T is the thermodynamic temperature, and n is the reduction reaction transfer coefficient.

The first term on the right side of (2) is the overpotential caused by electrochemical polarization, and the second term is the overpotential caused by concentration polarization. The values of J_0_ and J_d_ are constant for a particular reaction system. When J_K_ approaches J_d_, the overpotential caused by concentration polarization is large, and the diffusion step becomes the control step. At this time, the low duty ratio means the on-time is short, the off-time is long. During the off time, the ions near the surface of the cathode diffuse sufficiently, which is beneficial to reduce the concentration polarization, reduce the thickness of the diffusion layer, and facilitate the obtaining of the fine electroformed layer. When the values of J_K_ and J_d_ are not very close, the second term has a small overpotential caused by concentration polarization, and the first electrochemical polarization overpotential is large. At this time, the electron transfer step becomes a control step. If the duty ratio is too small, the average current density will be caused, the electrochemical polarization is reduced, and the influence of pulse electroforming to refine the grains is not obvious, so the choice of duty ratio is very important for the pulse electroforming process.

**Figure 11:**
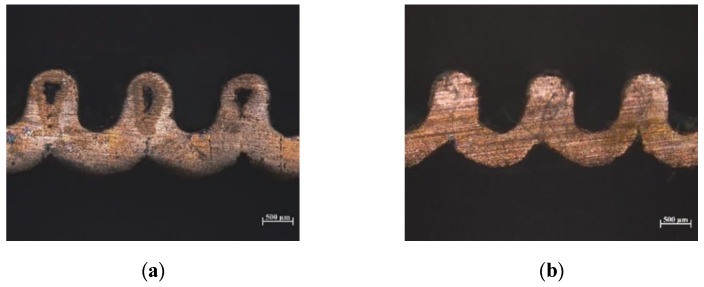
Cross-section of micro-grooves. (**a**) Electroformed micro-grooves with direct current and (**b**) electroformed micro-grooves with pulse current.

Taking micro-grooves as an example for comparison experiment, as shown in Figure 11a, the cross-section of micro-grooves obtained when the direct current density was 6 A/dm^2^, and Figure 11b shows micro-grooves fabricated with a peak current density of 6 A/dm^2^ and a duty ratio of 20%. It can be seen from the Figure 11 that the thickness of the micro-grooves electroformed layer made by direct current was not uniform, which was due to the uneven distribution of the current density on the cathode surface. Due to the higher current density, the top and sidewall grew too fast during the deposition process, while the bottom grew too slowly to form a microgroove with an uneven electroformed layer. As the electroforming time was lengthened, voids as shown in Figure 11a, were gradually formed between the micro grooves. Compared with the direct current, there was a pulse intermittent time during electroforming process with pulse current, which reduced the concentration polarization, and grain nucleation rate was faster than the growth rate. It is beneficial to obtain a fine and uniform electroformed layer. Therefore, the pulse current could improve the unevenness of the electroformed layer.

**Figure 12:**
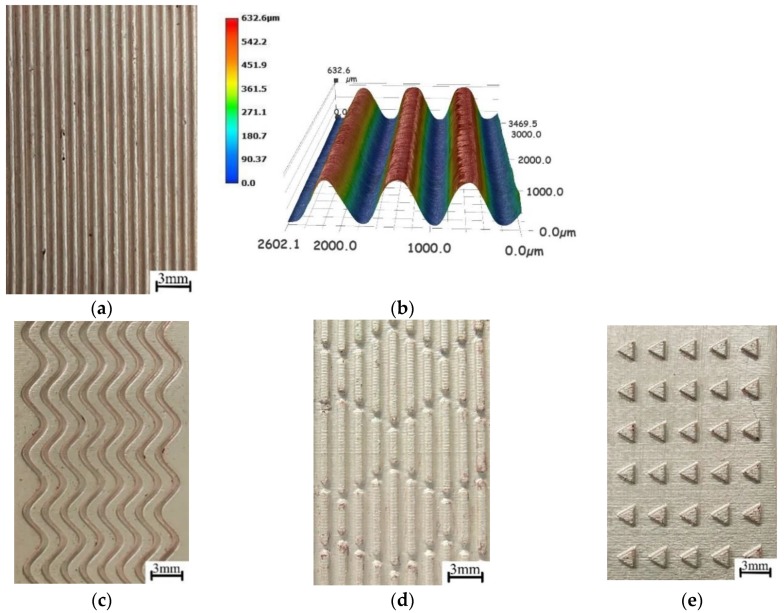
Three-dimensional microstructures. (**a**) Three-dimensional micro-grooves; (**b**) three-dimensional topography of micro-grooves; (**c**) curved micro-grooves; (**d**) sharkskin surface microstructure; and (**e**) triangular microstructure.

The electroforming experiment was carried out on different morphological microstructures with a peak current density of 6 A/dm^2^, a duty cycle of 20%, and a distance between electrodes of 40 mm. The microstructures fabricated by the electroforming experiment and three-dimensional topography of micro-grooves are shown in Figure 12.

## 4. Conclusions

In this paper, a fabrication method of a three-dimensional metal microstructure was presented by combining 3D printing technology and electroforming technology. Through experimental analysis, the current density and distance between electrodes had a certain influence on the surface morphology and microhardness of the electroformed layer. The use of pulse current for electroforming experiment could improve the quality and uniformity of micro-groove forming and improve the cavity generated by DC current electroforming. The uniform, voidless micro-grooves could be fabricated with the peak current density of 6 A/dm^2^, the duty ratio of 20%, and the distance between electrodes of 40 mm.

## Figures and Tables

**Table 1:** Composition of the electroformed copper electrolyte and condition.

Composition of the Solution or Process Conditions	Value or Condition
Copper (II) sulfate pentahydrate (Cu_2_SO_4_·5H_2_SO_4_)	200 g/L
Concentrated sulfuric acid (H_2_SO_4_)	60 g/L
PH	1
Temperature	26 °C
